# Diagnostic accuracy of SSR-PET/CT compared to histopathology in the identification of liver metastases from well-differentiated neuroendocrine tumors

**DOI:** 10.1186/s40644-023-00614-2

**Published:** 2023-09-28

**Authors:** M. P. Fabritius, V. Soltani, C. C. Cyran, J. Ricke, P. Bartenstein, C. J. Auernhammer, C. Spitzweg, M. L. Schnitzer, R. Ebner, S. Mansournia, A. Hinterberger, A. Lohse, G. T. Sheikh, M. Winkelmann, T. Knösel, M. Ingenerf, C. Schmid-Tannwald, W. G. Kunz, J. Rübenthaler, Freba Grawe

**Affiliations:** 1 Department of Radiology, University Hospital, LMU Munich, Marchioninistr. 15, 81377 Munich, Germany; 2 Department of Nuclear Medicine, University Hospital, LMU Munich, 81377 Munich, Germany; 3 Department of Internal Medicine 4, University Hospital, LMU Munich, 81377 Munich, Germany; 4 Interdisciplinary Center of Neuroendocrine Tumors of the GastroEnteroPancreatic System (GEPNET-KUM, ENETS certified Center of Excellence), University Hospital, LMU Munich, 81377 Munich, Germany; 5 Department of Pathology, University Hospital, LMU Munich, 81377 Munich, Germany

**Keywords:** NET, SSR, PET/CT, MRI, Liver metastases

## Abstract

**Background:**

Histopathology is the reference standard for diagnosing liver metastases of neuroendocrine tumors (NETs). Somatostatin receptor-positron emission tomography / computed tomography (SSR-PET/CT) has emerged as a promising non-invasive imaging modality for staging NETs. We aimed to assess the diagnostic accuracy of SSR-PET/CT in the identification of liver metastases in patients with proven NETs compared to histopathology.

**Methods:**

Histopathologic reports of 139 resected or biopsied liver lesions of patients with known NET were correlated with matching SSR-PET/CTs and the positive/negative predictive value (PPV/NPV), sensitivity, specificity, and diagnostic accuracy of SSR-PET/CT were evaluated. PET/CT reading was performed by one expert reader blinded to histopathology and clinical data.

**Results:**

133 of 139 (95.7%) liver lesions showed malignant SSR-uptake in PET/CT while initial histopathology reported on ‘liver metastases of NET´ in 127 (91.4%) cases, giving a PPV of 91.0%. Re-biopsy of the initially histopathologically negative lesions (reference standard) nevertheless diagnosed ‘liver metastases of NET’ in 6 cases, improving the PPV of PET/CT to 95.5%. Reasons for initial false-negative histopathology were inadequate sampling in the sense of non-target biopsies. The 6 (4.3%) SSR-negative lesions were all G2 NETs with a Ki-67 between 2–15%.

**Conclusion:**

SSR-PET/CT is a highly accurate imaging modality for the diagnosis of liver metastases in patients with proven NETs. However, we found that due to the well-known tumor heterogeneity of NETs, specifically in G2 NETs approximately 4–5% are SSR-negative and may require additional imaging with [^18^F]FDG PET/CT.

**Supplementary Information:**

The online version contains supplementary material available at 10.1186/s40644-023-00614-2.

## Background

Neuroendocrine tumors (NETs) arise from cells of the endocrine system found throughout the body and occur most commonly in the gastrointestinal tract, pancreas and lungs [1, 2]. Due to their rarity and heterogeneity, diagnosis and management of NETs can be challenging [3–5]. Since the liver represents the most common site for metastatic disease in NETs and is associated with significant morbidity and mortality, early diagnosis and accurate staging is essential for optimal patient management and improved outcome [6–8].

Historically, histopathology has been the reference standard for diagnosing liver metastases of NETs. However, this approach has limitations, including potential sampling errors and the inability to assess the entire liver tissue. Moreover, biopsy procedures are invasive and costly. In recent years, somatostatin receptor positron emission tomography/computed tomography (SSR-PET/CT) has emerged as the guideline-recommended non-invasive imaging modality for diagnosing and staging of highly differentiated NETs. SSR-PET/CT uses radiolabeled SSR-analogs, which bind to SSR that are highly expressed in NETs. According to the World Health Organization (WHO), tumor classification of NETs of the gastroenteropancreatic system is based on the morphology and proliferation index (Ki-67) into grade 1–3 [9]. Grade 1 (Ki-67 < 2%) and grade 2 (Ki-67 3-20%) NETs have high somatostatin receptor (SSR) expression, whereas grade 3 (Ki-67 > 20%) NETs show higher glucose metabolism and lower SSR-expression [6, 10].

The aim of this study was to compare the diagnostic accuracy of SSR-PET/CT in the identification of liver metastases of NET patients in a lesion-based manner with histopathology as the reference standard.

## Methods

### Study design and patient cohort

**Fig. 1:**
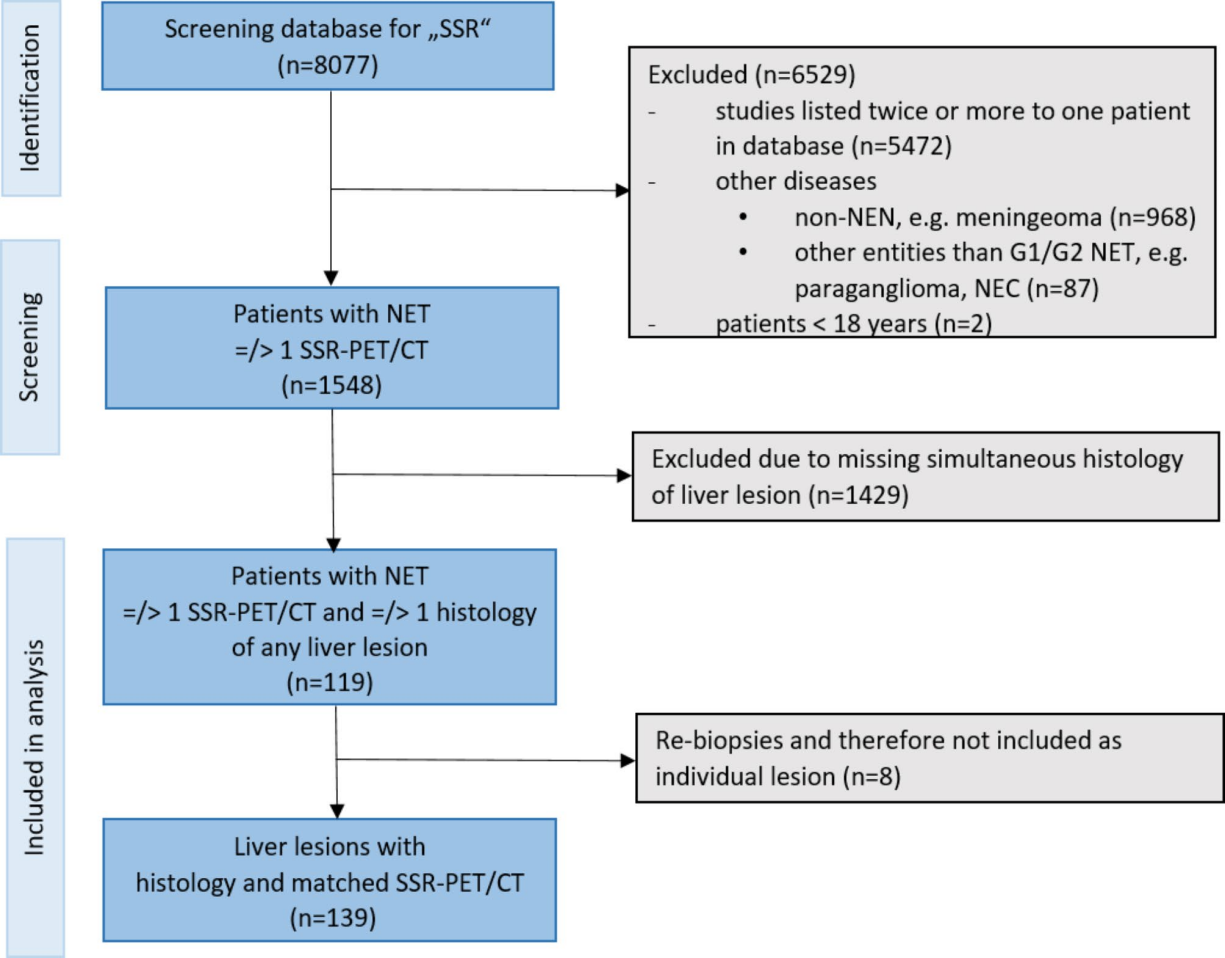
Flow chart of patient selection

Our institutional database was screened retrospectively for the term SSR imaging studies between 2006 and 2021 and a total of 8077 results were found. These imaging studies were assigned to a total of 2605 patients. We excluded all studies listed multiple times to one patient, studies of patients < 18 years or of other entities than G1/G2 NETs of the gastroenteropancreatic and bronchopulmonary tract (e.g., meningioma, paraganglioma) in the further analysis. Of the therefore remaining 1584 patients, 1429 patients without histopathology of the liver were excluded. Thus 119 patients with differentiated NET (grade 1/2) with a total of 139 lesions with correlating histopathology (obtained within a maximum of 6 months) were included for the final analysis. A flow chart of the patient selection is presented in Fig. 1. The reference standard was defined as histopathology after re-biopsy in cases of initially negative results due to assessment of non-target lesions. Additionally, results prior to re-biopsy are presented for a better overview of non-target biopsies. Histopathology was obtained by CT- or ultrasound-guided biopsy in 67% (n = 92), surgical resection in 32% (n = 45) and hemihepatectomy in 1% (n = 2) of the cases. The reference standard was compared in a lesion-based analysis of the correlating 139 SSR-PET/CTs by one expert reader (> 10 years of experience) by labeling all liver lesions with malignant SSR-uptake consistent with NET metastasis, blinded to histopathology and clinical data. The index lesion was defined as the lesion which was biopsied and correlated to SSR-PET/CT. In cases where the expert did not label the index lesion on PET/CT as a NET metastasis (n = 6), the expert was unblinded and the PET/CT was reviewed together with additional available information, in particular with a generally available corresponding liver MRI.

### SSR-PET/CT imaging

SSR-PET/CT scans were acquired on a Biograph 64 TruePoint w/TrueV and Biograph mCT Flow 20-4R PET/CT scanner (Siemens Healthineers, Erlangen, Germany). PET/CT scans were initiated 67 ± 11 min after administration of 218 ± 33 MBq [68Ga]Ga-DOTA-TATE PET/CT and 62 ± 10 min after administration of 231 ± 20 MBq [68Ga]Ga-DOTATOC. Data on tracer administration was not available in one case. After intravenous injection of 1.5 times the body weight of contrast agent (Ultravist 300, Bayer Vital GmbH, Leverkusen, Germany or Imeron 350 mg/mL, 2.5 ml/s, Bracco Imaging Deutschland GmbH) diagnostic venous-phase CT scans of the neck, thorax, abdomen, and pelvis (100–190 mAs; 120 kV) were acquired. Patients received diagnostic CT scans without contrast enhancement in cases of known severe allergic reactions to iodinated contrast agent, renal impairment/failure, or hyperthyroidism. Image construction was automatically performed using built-in software. 3 mm-slice reconstructions were used for reading. SSR-expression in PET/CT was assessed visually using the Krenning score.

### Statistical analysis

All continuous variables were expressed as mean and standard deviation (SD). For statistical analysis, diagnostic accuracy of SSR-PET/CT was tested using positive predictive value (PPV), negative predictive value (NPV), sensitivity and specificity. Additionally, exact 95% confidence intervals (CI) were calculated for all values.

## Results

### Patient characteristics

Patient characteristics are summarized in Table 1. Biopsy led to the diagnosis of NET in 33/139 (24%) of cases. The remaining 106/139 (76%) biopsies were performed for molecular analysis or exclusion of secondary malignancies.

**Table 1:** Patient characteristics

Patient characteristics	n (%)
**Sex**	
Female	47 (39%)
Male	72 (61%)
**Location of primary**	
Gastroenteropancreatic	104 (87)
Pancreas	43 (41)
Small intestine	49 (47)
Colon	10 (10)
Liver	2 (2)
Lung	10 (8)
CUP	4 (3)
Kidney	1 (1)
**SSR-analogs**	
[^68^Ga]Ga-DOTATOC	89 (64%)
[^68^Ga]Ga-DOTA-TATE	50 (36%)

SSR, somatostatin receptor.

Mean age ± SD was 63.0 ± 12.3 years

### Diagnostic accuracy of PET/CT in the detection of liver metastases

133 of 139 (95.7%) liver lesions were detected and labeled as NET metastases with malignant SSR-expression. The reference standard histopathology after re-biopsy of initially negative results reported on ‘liver metastases of NET’ in 127 of 139 (91.4%) cases of which 34 (27%) were metastases of G1 NET and 93 (73%) of G2 NET, resulting in a PPV of 95.5%. Before re-biopsy, the PPV of PET/CT was 91.0%. Hepatic involvement detected by SSR-PET/CT and histopathology after first biopsy and re-biopsy is presented in Tables 2 and 3. An overview of all results (PPV, NPV, sensitivity and specificity for PET/CT vs. histopathology before and after re-biopsy is shown in Table 4.

**Table 2:** Hepatic involvement detected by SSR-PET/CT and histopathology after first biopsy

	Histopathology		
**SSR-PET/CT**	Positive	Negative	n_total_
Positive	121	12	133
Negative	6	0	6
n_total_	127	12	139

.

**Table 3:** Hepatic involvement detected by SSR-PET/CT and histopathology after re-biopsy

	Histopathology		
**SSR-PET/CT**	Positive	Negative	n_total_
Positive	127	6	133
Negative	6	0	6
n_total_	133	6	139

.

**Table 4:** PPV, NPV, sensitivity, specificity and diagnostic accuracy for SSR-PET/CT. PPV, positive predictive value; NPV, negative predictive value.

	Biopsy	Re-biopsy (reference standard)
PPV	91.0% (95%CI: 84.8%, 95.3%)	95.5% (95%CI: 90.4%, 98.3%)
NPV	0% (95%CI: 0%, 45.9%)	0% (95%CI: 0%, 45.9%)
Sensitivity	95.2% (95%CI: 90.0%, 98.3%)	95.5% (95%CI: 90.4%, 98.3%)
Specificity	0% (95%CI: 0%, 26.5%)	0% (95%CI: 0%, 45.9%)
Accuracy	87.1% (95%CI: 80.0%, 92.1%)	91.4% (95%CI: 85.4%, 95.5%)

PPV, positive predictive value; NPV, negative predictive value

NPV and Specificity are both recorded as 0% due to the deliberate study design focusing on a patient cohort with a notably high prevalence of liver metastases.

Additionally, diagnostic accuracy was calculated for G1 and G2 NETs separately and showed similar excellent results with a PPV of 100%, respectively. An overview on all results (PPV, NPV, sensitivity, specificity, and diagnostic accuracy) are presented in the supplements in Tabe S1 and S2.

### SSR-positive liver lesions without histopathological correlate

**Fig. 2:**
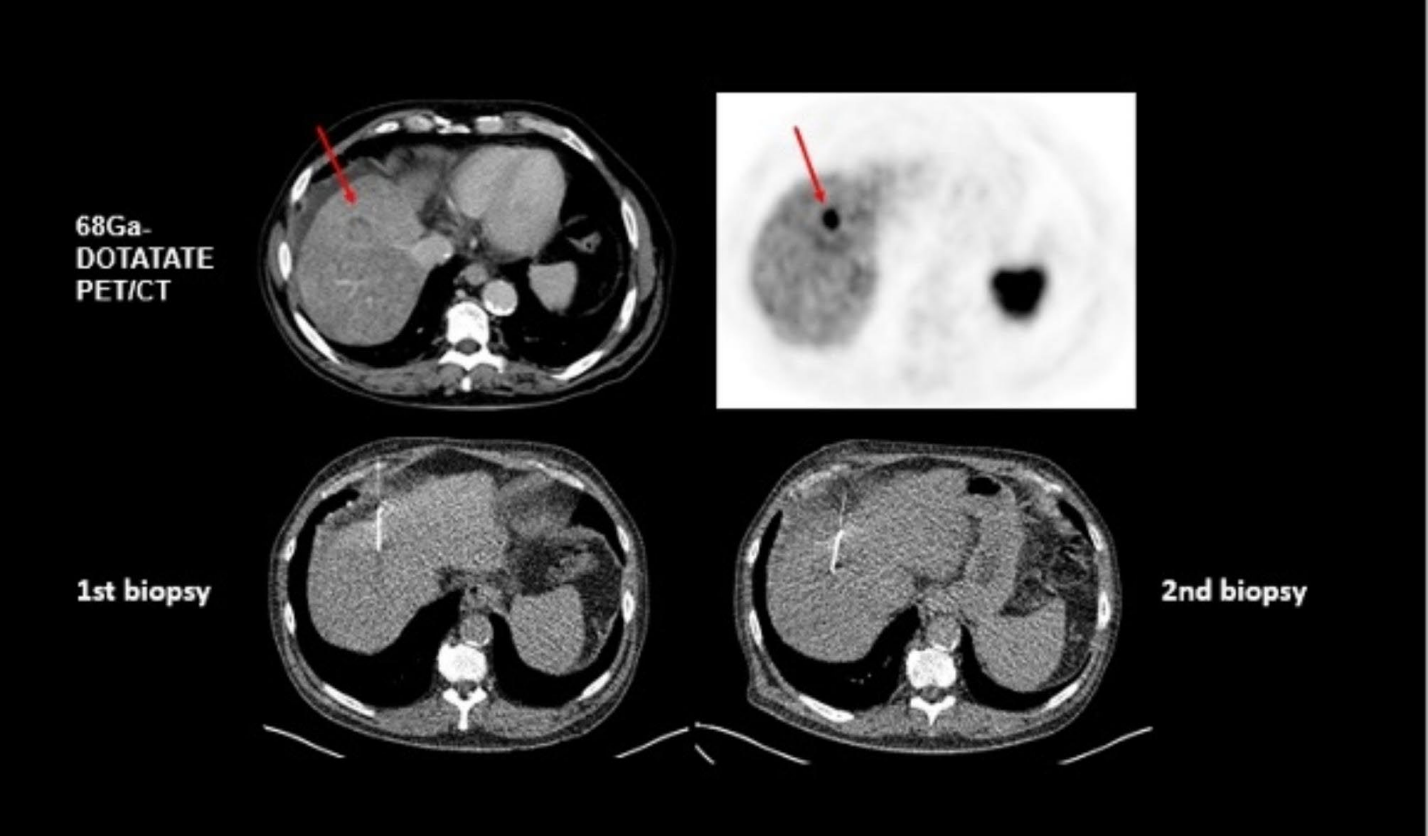
Initially false-negative histopathological result of a liver metastasis in a 64 year old patient with a newly diagnosed NET G1 (Ki-67 2%) of the pancreas. On CT (first row, left image, venous phase), hepatic metastasis in segment 8 with 2.8 × 2.8 cm was clearly delineated with enhanced [^68^Ga]Ga-DOTA TATE-uptake in PET (first row, right image). First biopsy was negative, whereas second biopsy confirmed liver metastasis of NET G1 on histopathological examination (second row, both images)

In 12 cases, all of which were marked by the reader as NET metastases on PET/CT, no tumor tissue could be detected by histopathology. A re-biopsy was performed in 8 of 12 cases: in 6 of those cases, liver metastases of a NET (G1/2) were finally diagnosed, whereas 2 of the re-biopsies confirmed negative histopathological results. In one case, the negative histopathological result was confirmed by a further re-biopsy, whereas the second case was not further followed. A patient example is presented in Fig. 2. Thus, a low rate of false-positive PET findings seems possible.

### Histopathologically confirmed liver metastases of NET without correlate in SSR-PET/CT

In 6 (4.3%) cases, liver metastases of NET were reported by histopathology, but the corresponding lesions did not show any SSR expression in PET/CT.

**Fig. 3:**
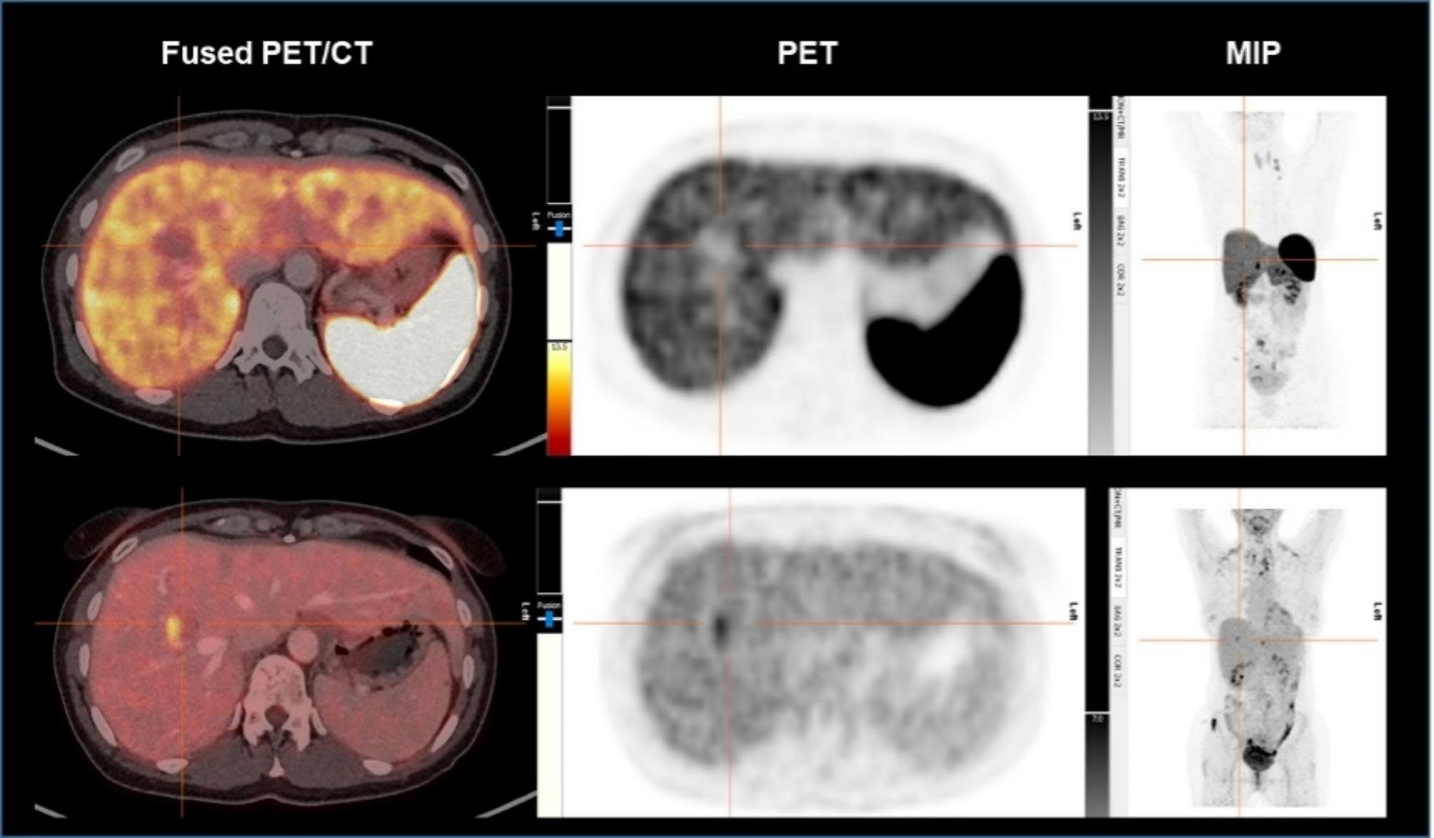
PET/CT of a 47 year old patient with a NET G2 (Ki-67 8%) of the small intestine. Liver lesion in segment 8 with 2.8 × 2.7 cm without [^68^Ga]Ga-DOTATOC-uptake in SSR-PET/CT (first row). [^68^Ga]Ga-DOTATOC-uptake of the small intestine (primary site) led to the suspicion of undifferentiated liver metastases. Additional [^18^F]FDG PET/CT was performed and showed enhanced [^18^F]FDG-uptake (SUVmax 5.8) compared to liver parenchyma. Left images show fused PET/CT (first row: [^68^Ga]Ga-DOTATOC PET/CT, second row: [^18^F]FDG PET/CT, middle images show PET; right images show maximum intensity projections (MIP).

Two patients with NET of the small intestine (Ki-67 2%, 3.4 × 2.6 cm and Ki-67 10%, 1.6 × 1.3 cm) showed SSR-negative liver metastases but were [^18^F]FDG-positive in additional [^18^F]FDG PET/CT (SUVmax 4.9 and SUVmax 8.9). One patient showed SSR-uptake of the primary tumor in the small intestine but was SSR-negative for liver (Ki-67 8%) and histologically confirmed ovarian metastases, which again were positive in additional [^18^F]FDG PET/CT (2.8 × 2.7 cm, SUVmax 5.8; see Fig. 3). One patient with a NET of the small intestine (Ki-67 5%) received radioembolization of the liver metastases and was SSR-negative, whereas MRI of the liver showed residual arterial hypervascularization (7.5 × 5.4 cm). Another patient with a carcinoid of the lung (Ki-67 15%, 2.4 × 1.6 cm) and one patient with a NET of the pancreas (Ki-67 5%, 2.4 × 2.0 cm) showed SSR-negative liver metastases. Table 5 gives an overview on the results of the histopathological examinations of those lesions.

**Table 5:** Histopathological results of the 6 liver lesions without SSR-uptake in PET/CT

Lesion	Primary of NET	Liver metastases
**1**	Small intestine, pT3, pN1 (6/14), L1, V0, local R0, pM1, G2, Ki-67 > 2%	G1, Ki-67 2%
**2**	Small intestine, pT3, pN1 (1/13), L1, V1, Pn1, R1 (liver), pM1, G2, Ki-67 1–15%,	G2, Ki-67 10%
**3**	Small intestine, pT4, pN2 (13 / 29), pM1c (HEP, PER, OTH), L1, V0, Pn1, R1	G2, Ki-67 8%
**4**	Small intestine, pT2, pN1 (4/31) L0, V0, G2, Ki-67 3–4%	G2, Ki-67 5%
**5**	Pancreas, pT2, pN0, L0, V0, G1, R0, cM0	G2, Ki-67 5%
**6**	Carcinoid of the lung, histopathological report on primary not available	G2, Ki-67 15%

.

## Discussion

In this retrospective study, we performed a liver lesion-based comparison of histopathologic reports and SSR-PET/CT ([^68^Ga]Ga-DOTATOC and [^68^Ga]Ga-DOTA-TATE) findings in patients with diagnosed NET. Our results demonstrate high diagnostic accuracy of SSR-PET/CT in the identification of liver metastases of NET patients with an excellent PPV of 91.0% and an even higher PPV of 95.5% when compared to re-biopsy of initially negative histopathological results.

Several previous studies have demonstrated the high diagnostic accuracy of SSR-PET/CT in the evaluation of NETs. In a large study of 728 NET patients, [^68^Ga]Ga-DOTA-TATE PET/CT showed high sensitivity, specificity, accuracy, positive predictive value, and negative predictive value (97.0%, 95.1%, 96.6%, 98.5%, and 90.4%) in the detection of overall NET lesions [11]. Specifically identifying hepatic involvement in NET patients, similar high diagnostic accuracy was reported for SSR-PET/CT compared to the reference standard liver MRI with a sensitivity of 97.0% and a specificity of 97.7%, a PPV of 99.0% and NPV of 93.0% [12]. Moreover, SSR-PET/CT was found to have an important value in the diagnosis of pulmonary neuroendocrine tumors (carcinoids) and its distant metastases [13, 14]. However, one patient in our cohort with a carcinoid of the lung and a Ki-67 of 15% (G2) showed SSR-negative liver metastases. *Haug et al. also reported on low tracer of liver metastases in a patient with atypical carcinoid tumor of intermediate grade* [15].

In 12 (8.6%) SSR-positive lesions, no tumor tissue was detected by initial histopathology in our cohort, whereas tumor cells of NETs were identified after re-biopsy in most cases (6/8 cases). Thus, the underlying reasons were most likely inadequate sampling in the sense of non-target biopsies. This finding emphasizes that the absence of detectable metastatic lesions on liver biopsy does not necessarily rule out the presence of liver metastases of NET patients. Tumor heterogeneity of NETs is well known, and low Ki-67 may be challenging to be detected on histological examinations. Moreover, not all liver metastases are easily accessible for biopsies, for example if located close to large vessels. In consideration of these possible false-negative biopsy results, histologic verification of PET-positive liver lesions appears redundant in cases of patients with histologically proven NETs, and functional imaging with SSR-PET/CT might reduce unnecessary further biopsies. However, one must be aware that biopsies are nowadays not only used for pure histological confirmation of metastases but are also required for additional molecular diagnostics in precision medicine [16].

In 6 (4.3%) cases, *the index lesion was not labeled as NET metastasis in SSR-PET/CT, however, subsequent dynamic, contrast-enhanced MRI of the liver unequivocally confirmed these lesions as metastatic NETs. This finding emphasizes the ongoing essential role of liver MRI in specific cases. Notably, all 6 of these lesions were categorized as G2 in the corresponding histopathological reports, with Ki-67 values ranging between 2% and 15%. The observed heterogeneity of SSR-expression patterns in NETs is likely attributed to the inherent tumor/metastatic variability, resulting in diverse SSR-expression profiles even within the same patient across different lesions* [17, 18]. Furthermore, it is well known that NETs of low differentiation lack SSR-expression and SSR-PET/CT has low sensitivity but can be localized by [^18^F]FDG PET/CT [2, 19, 20]. Liver metastases from NETs are often diagnosed by CT- or ultrasound-guided biopsy, in which tumor tissue is obtained randomly from a specific lesion, depending on accessibility, and the Ki-67 is determined without targeting regions that may have a higher proliferation rate [21]. In our analysis, in 2 patients with NET G2 (Ki-67 2% and Ki-67 10%, respectively) additional [^18^F]FDG PET/CT was performed and showed high [^18^F]FDG-uptake indicating intertumoral heterogeneity with tumor cells of lower differentiation. For these cases, a proposed grading scheme which describes the joint results of both, the [^18^F]FDG- and SSR-PET scans in dual tracer imaging (NETPET), showed promising results as a prognostic biomarker and correlated significantly with overall survival [1, 22]. Therefore, [^18^F]FDG PET/CT may have a clinical role in intermediate grade neuroendocrine neoplasms based on clinical presentation (e.g., patients with CT progression or with SSR-negative lesions). Despite above mentioned limitations in a small number of patients, SSR-PET/CT represents a whole-body, one-stop-shop diagnostic tool for NETs.

*There are various limitations to this study. Firstly, this was a retrospectively conducted single-center study. The use of SSR-analogues ([*^*68*^*Ga]Ga-DOTATOC and [*^*68*^*Ga]Ga-DOTA-TATE) varied across patients and examinations. Nevertheless, previous studies have consistently reported no differences in diagnostic accuracy when evaluating sensitivities and uptake values among different SSR-ligands, including considerations of tumor origin and grading* [23, 24]. *Additionally, the effective doses of both radiopharmaceuticals are comparable, rendering them equivalent even from a radiation dosimetry perspective* [25]. *Furthermore, not all patients with SSR-negative NET lesions received additional [*^*18*^*F]FDG PET/CT for further diagnostic evaluation. The lack of [*^*18*^*F]FDG PET/CT data for some SSR-negative patients could potentially influence the overall diagnostic accuracy and completeness of our findings. Although we have not directly compared the diagnostic performance of SSR imaging and [*^*18*^*F]FDG PET/CT in this study, acknowledging this limitation is essential for a comprehensive understanding of the scope and implications of our results. Future studies that incorporate both SSR imaging and [*^*18*^*F]FDG PET/CT data for all patient groups could offer a more comprehensive assessment of the diagnostic capabilities of these imaging modalities in the context of SSR-negative NET lesions.*

## Conclusion

In conclusion, SSR-PET/CT is a highly accurate imaging modality for the diagnosis of liver metastases of NET patients. However, due to the well-known tumor heterogeneity of NETs, specifically 4–5% of G2 NETs are SSR-negative and additional imaging with [^18^F]FDG PET/CT or functional imaging-based biopsy is useful to identify higher-grade disease.

### Electronic supplementary material

Below is the link to the electronic supplementary material.

Supplementary Material 1: Table S1 PPV, NPV, sensitivity, specificity and diagnostic accuracy for SSR-PET/CT in NET G1 patients. PPV, positive predictive value; NPV, negative predictive value.

Supplementary Material 2: Table S2 PPV, NPV, sensitivity, specificity and diagnostic accuracy for SSR-PET/CT in NET G2 patients. PPV, positive predictive value; NPV, negative predictive value.

## Data Availability

The data that support the findings of this study are available from the corresponding author upon reasonable request.

## Abbreviations

NET: Neuroendocrine tumor
>SSR: Somatostatin receptor
PET/CT: Positron emission tomography/computed tomography
G: Grade
FDG: Fluorodeoxyglucose
WHO: World Health Organization
DOTA-TOC: DOTA(0)-Phe(1)-Tyr(3))octreotide
DOTA-TATE: DOTA-0-Tyr3-Octreotate
SD: Standard deviation
PPV: Positive predictive value
CI: Confidence intervals

## References

[1] Chan DL, Pavlakis N, Schembri GP, Bernard EJ, Hsiao E, Hayes A (2017). Dual somatostatin Receptor/FDG PET/CT imaging in metastatic neuroendocrine tumours: proposal for a Novel Grading Scheme with Prognostic significance. Theranostics.

[2] Raphael MJ, Chan DL, Law C, Singh S (2017). Principles of diagnosis and management of neuroendocrine tumours. CMAJ.

[3] Dasari A, Shen C, Halperin D, Zhao B, Zhou S, Xu Y (2017). Trends in the incidence, prevalence, and survival outcomes in patients with neuroendocrine tumors in the United States. JAMA Oncol.

[4] Kulke M, Benson A, Bergsland E, Berlin J, Blaszkowsky L, Choti M (2012). Neuroendocrine Tumors Journal of the National Comprehensive Cancer Network.

[5] Bodei L, Ambrosini V, Herrmann K, Modlin I (2017). Current concepts in (68)Ga-DOTATATE Imaging of Neuroendocrine Neoplasms: Interpretation, Biodistribution, Dosimetry, and molecular strategies. J Nucl Med.

[6] Rinke A, Müller HH, Schade-Brittinger C, Klose KJ, Barth P, Wied M (2009). Placebo-controlled, double-blind, prospective, randomized study on the effect of octreotide LAR in the control of tumor growth in patients with metastatic neuroendocrine midgut tumors: a report from the PROMID Study Group. J Clin Oncol.

[7] Swiha MM, Sutherland DEK, Sistani G, Khatami A, Abazid RM, Mujoomdar A (2022). Survival predictors of (177)Lu-Dotatate peptide receptor radionuclide therapy (PRRT) in patients with progressive well-differentiated neuroendocrine tumors (NETS). J Cancer Res Clin Oncol.

[8] Panzuto F, Nasoni S, Falconi M, Corleto VD, Capurso G, Cassetta S (2005). Prognostic factors and survival in endocrine tumor patients: comparison between gastrointestinal and pancreatic localization. Endocr Relat Cancer.

[9] Nagtegaal ID, Odze RD, Klimstra D, Paradis V, Rugge M, Schirmacher P (2020). The 2019 WHO classification of tumours of the digestive system. Histopathology.

[10] Rufini V, Baum RP, Castaldi P, Treglia G, De Gaetano AM, Carreras C (2012). Role of PET/CT in the functional imaging of endocrine pancreatic tumors. Abdom Imaging.

[11] Skoura E, Michopoulou S, Mohmaduvesh M, Panagiotidis E, Al Harbi M, Toumpanakis C (2016). The impact of 68Ga-DOTATATE PET/CT imaging on management of patients with neuroendocrine tumors: experience from a National Referral Center in the United Kingdom. J Nucl Med.

[12] Grawe F, Rosenberger N, Ingenerf M, Beyer L, Eschbach R, Todica A (2023). Diagnostic performance of PET/CT in the detection of liver metastases in well-differentiated NETs. Cancer Imaging.

[13] Rufini V, Lorusso M, Inzani F, Pasciuto T, Triumbari EKA, Grillo LR (2022). Correlation of somatostatin receptor PET/CT imaging features and immunohistochemistry in neuroendocrine tumors of the lung: a retrospective observational study. Eur J Nucl Med Mol Imaging.

[14] Deleu AL, Laenen A, Decaluwé H, Weynand B, Dooms C, De Wever W (2022). Value of [(68)Ga]Ga-somatostatin receptor PET/CT in the grading of pulmonary neuroendocrine (carcinoid) tumours and the detection of disseminated disease: single-centre pathology-based analysis and review of the literature. EJNMMI Res.

[15] Haug AR, Cindea-Drimus R, Auernhammer CJ, Reincke M, Wängler B, Uebleis C (2012). The role of 68Ga-DOTATATE PET/CT in suspected neuroendocrine tumors. J Nucl Med.

[16] Boilève A, Faron M, Fodil-Cherif S, Bayle A, Lamartina L, Planchard D (2023). Molecular profiling and target actionability for precision medicine in neuroendocrine neoplasms: real-world data. Eur J Cancer.

[17] Nilica B, Waitz D, Stevanovic V, Uprimny C, Kendler D, Buxbaum S (2016). Direct comparison of (68)Ga-DOTA-TOC and (18)F-FDG PET/CT in the follow-up of patients with neuroendocrine tumour treated with the first full peptide receptor radionuclide therapy cycle. Eur J Nucl Med Mol Imaging.

[18] Reccia I, Pai M, Kumar J, Spalding D, Frilling A. Tumour Heterogeneity and the consequent practical Challenges in the management of gastroenteropancreatic neuroendocrine neoplasms. Cancers (Basel). 2023;15. 10.3390/cancers15061861. 10.3390/cancers15061861 PMC10047148 36980746

[19] Zhang P, Yu J, Li J, Shen L, Li N, Zhu H (2018). Clinical and prognostic value of PET/CT imaging with combination of (68)Ga-DOTATATE and (18)F-FDG in gastroenteropancreatic neuroendocrine neoplasms. Contrast Media Mol Imaging.

[20] Kaemmerer D, Peter L, Lupp A, Schulz S, Sänger J, Prasad V (2011). Molecular imaging with Ga-SSTR PET/CT and correlation to immunohistochemistry of somatostatin receptors in neuroendocrine tumours. Eur J Nucl Med Mol Imaging.

[21] Yang Z, Tang LH, Klimstra DS (2011). Effect of tumor heterogeneity on the assessment of Ki67 labeling index in well-differentiated neuroendocrine tumors metastatic to the liver: implications for prognostic stratification. Am J Surg Pathol.

[22] Chan DL, Hayes AR, Karfis I, Conner A, Furtado O’Mahony L, Mileva M (2023). Dual [(68)Ga]DOTATATE and [(18)F]FDG PET/CT in patients with metastatic gastroenteropancreatic neuroendocrine neoplasms: a multicentre validation of the NETPET score. Br J Cancer.

[23] Poeppel TD, Binse I, Petersenn S, Lahner H, Schott M, Antoch G (2011). 68Ga-DOTATOC versus 68Ga-DOTATATE PET/CT in functional imaging of neuroendocrine tumors. J Nucl Med.

[24] Kabasakal L, Demirci E, Ocak M, Decristoforo C, Araman A, Ozsoy Y (2012). Comparison of Ga-DOTATATE and Ga-DOTANOC PET/CT imaging in the same patient group with neuroendocrine tumours. Eur J Nucl Med Mol Imaging.

[25] Sandström M, Velikyan I, Garske-Román U, Sörensen J, Eriksson B, Granberg D (2013). Comparative biodistribution and radiation dosimetry of 68Ga-DOTATOC and 68Ga-DOTATATE in patients with neuroendocrine tumors. J Nucl Med.

