# Supplementary material for: Diagnostic accuracy of SSR-PET/CT compared to histopathology in the identification of liver metastases from well-differentiated neuroendocrine tumors
Source: Cancer Imaging. 2023 Sep 28;23:92. doi: 10.1186/s40644-023-00614-2 (PMC10537814; doi:10.1186/s40644-023-00614-2)
Supplement: Supplementary file 1 — Supplementary Material 1: Table S1 PPV, NPV, sensitivity, specificity and diagnostic accuracy for SSR-PET/CT in NET G1 patients. PPV, positive predictive value; NPV, negative predictive value. [file 40644_2023_614_MOESM1_ESM.docx]

|  | Biopsy | **Re-biopsy (reference standard)** |
| --- | --- | --- |
| PPV | 97.0% (95%CI: 84.2%, 99.9%) | 100% (95%CI: 89.4%, 100%) |
| NPV | 0% (95%CI: 0%, 84.2%) | 0% (95%CI: 0%, 84.2%) |
| Sensitivity | 94.1% (95%CI: 80.3%, 99.3%) | 94.3% (95%CI: 80.8%, 99.3%) |
| Specificity | 0% (95%CI: 0%, 97.5%) | -- |
| Accuracy | 91.4% (95%CI: 76.9%, 98.2%) | 94.3% (95%CI: 80.8%, 99.3%) |
| PPV, positive predictive value; NPV, negative predictive value | | |
