# Supplementary material for: Diagnostic accuracy of SSR-PET/CT compared to histopathology in the identification of liver metastases from well-differentiated neuroendocrine tumors
Source: Cancer Imaging. 2023 Sep 28;23:92. doi: 10.1186/s40644-023-00614-2 (PMC10537814; doi:10.1186/s40644-023-00614-2)
Supplement: Supplementary file 2 — Supplementary Material 2: Table S2 PPV, NPV, sensitivity, specificity and diagnostic accuracy for SSR-PET/CT in NET G2 patients. PPV, positive predictive value; NPV, negative predictive value. [file 40644_2023_614_MOESM2_ESM.docx]

|  | Biopsy | **Re-biopsy (reference standard)** |
| --- | --- | --- |
| PPV | 98.9% (95%CI: 89.4%, 98.8%) | 100% (95%CI: 96.0%, 100%) |
| NPV | 0% (95%CI: 0%, 60.2%) | 0% (95%CI: 0%, 60.2%) |
| Sensitivity | 95.7% (95%CI: 89.4%, 98.8%) | 95.7% (95%CI: 89.5%, 98.8%) |
| Specificity | 0% (95%CI: 0%, 97.5%) | -- |
| Accuracy | 94.7% (95%CI: 88.0%, 98.3%) | 95.7% (95%CI: 89.4%, 98.8%) |
| PPV, positive predictive value; NPV, negative predictive value | | |
